# Postpartum Onset of Multiple Sclerosis: A Case Report

**DOI:** 10.7759/cureus.54972

**Published:** 2024-02-26

**Authors:** Catarina Afonso, Luís Bicheiro, Marta Lopes

**Affiliations:** 1 General Practice/Family Medicine, USF Planície, Évora, PRT; 2 General Practice/Family Medicine, USF Portas do Arade, Portimão, PRT

**Keywords:** case report, neurology, anxiety, postpartum, multiple sclerosis

## Abstract

A breastfeeding 29-year-old woman who gave birth to her first child three months ago presented at the family doctor’s appointment with a diverse array of symptoms, including vertigo, blurred vision, right lower limb weakness, and abdominal allodynia. She has a history of obesity and anxiety. The patient had a pre-pregnancy history of several visits to the family doctor. She takes folic acid, vitamin B12, iodine, and omega-3 supplements. The diverse symptomatology, the patient’s insistence on seeking medical care, and the history of anxiety pose significant hurdles in arriving at a timely diagnosis. Magnetic resonance imaging showed signs compatible with primary demyelinating lesions of the central nervous system, which elicited a referral to neurology. The clinical and imagiological findings were suggestive of multiple sclerosis (MS), for which a conservative approach was taken.

MS is a chronic inflammatory autoimmune disease with a mean age of onset of 20-30 years, more common in females. Stressful life events, viral infections, vaccination, physical trauma, previous anesthesia, excessive physical activity, and puerperium have all been described as trigger factors.

This case underscores the importance of vigilance in postpartum healthcare and the importance of conducting a comprehensive diagnostic assessment. Early diagnosis has a positive impact on the prognosis of the disease.

## Introduction

Multiple sclerosis (MS) is a chronic inflammatory autoimmune disease characterized by inflammation and neurodegeneration [1].

MS usually manifests in individuals in early adulthood, with an average age of onset ranging from 20 to 30 years. It can result in physical disability, cognitive challenges, and decreased quality of life [2].

The global prevalence of MS ranges from five to 300 cases per 100,000 people, with an increased occurrence observed at higher latitudes [2]. The overall life expectancy for individuals with MS is lower compared to the general population (75.9 years versus 83.4 years). MS affects more women than men, with a sex distribution of female to male ratio of almost 3:1 [2]. There is a notably higher risk of developing MS in Caucasian and African-American populations compared to Asian ones [3].

Among the factors that may trigger the first episode of MS and exacerbations are stressful events, viral infections, vaccination, physical trauma, anesthesia, excessive physical activity, and puerperium [4].

Stress is a very important trigger for the occurrence of both the first episode of illness and further exacerbations. Pregnancy reportedly has a much smaller role in these, having a favorable effect on relapses as pregnancy proceeds, while the postpartum period is associated with disease worsening [5,6].

## Case presentation

We report the case of a 29-year-old Caucasian woman who had recently given birth, G1P1L1 (G = gravidity; P = parity; L = live births), with a personal history of obesity and anxiety, without indication for chronic medication, taking folic acid, vitamin B12, iodine, and omega-3 supplementation. The pregnancy was monitored, and a healthy neonate was delivered at 39 weeks by c-section due to feto-pelvic incompatibility. She had a history of several visits to primary care for various complaints. In the immediate postpartum, she complained to her family doctor about mastalgia, breast engorgement, and difficulties in breastfeeding the newborn. This was resolved with pharmacological and non-pharmacological measures, resulting in exclusive breastfeeding for one month and mixed breastfeeding in the following months.

A week after giving birth, she further complained of constipation and light rectal bleeding, which were resolved with dietary changes and laxatives when needed. On examination, she also appeared anxious and easily irritable, having an Edinburgh Postnatal Depression Scale score of 9.

She returned for a medical appointment with her family doctor three months later, complaining of dizziness and nausea for the past month. She had, by this time, ceased all supplementations. She was discharged with betahistine 24 mg, BID, and metoclopramide 10 mg, OD, for one week.

One month later, she was feeling better, without nausea and only occasional vertigo. However, she complained of right leg discomfort and pain; the Homans sign was absent, and active and passive mobilization of the inferior limbs was preserved and painless. Blood work revealed no significant changes. Symptomatic treatment was proposed with tramadol 37.5 mg plus acetaminophen 325 mg, BID, for one week.

On revaluation with her family doctor, one week later, she reported a partial loss of strength in her right lower limb, which had been evolving for a week; also stated there had not been any pain reduction. Furthermore, she complained of constant head tremors and bilateral blurry vision for the past few days. The objective examination revealed resting tremors and reduced strength in the right lower limb, with no other alterations on neurological examination. Taking all of these into account, a contrast head computed tomography (CT) was scheduled, and a short course of nonsteroidal anti-inflammatory drugs (NSAIDs), together with propranolol 10 mg, BID, was prescribed.

She further sought medical attention one month later, complaining of fatigue, anxiety, and skin hypersensitivity in the abdominal area. On objective examination, she had allodynia in the abdominal area. The head-CT was inconclusive. In view of the persistence of a multitude of complaints since giving birth, as well as the multiple contacts with her doctor, anxiety and somatization disorder was put forward as a diagnostic hypothesis, but given the postpartum period, the patient’s age group, and the symptoms, the hypothesis of MS was also considered, making it necessary to request a cranial, cervical, dorsal, and lumbar magnetic resonance imaging (MRI).

**Figure 1:**
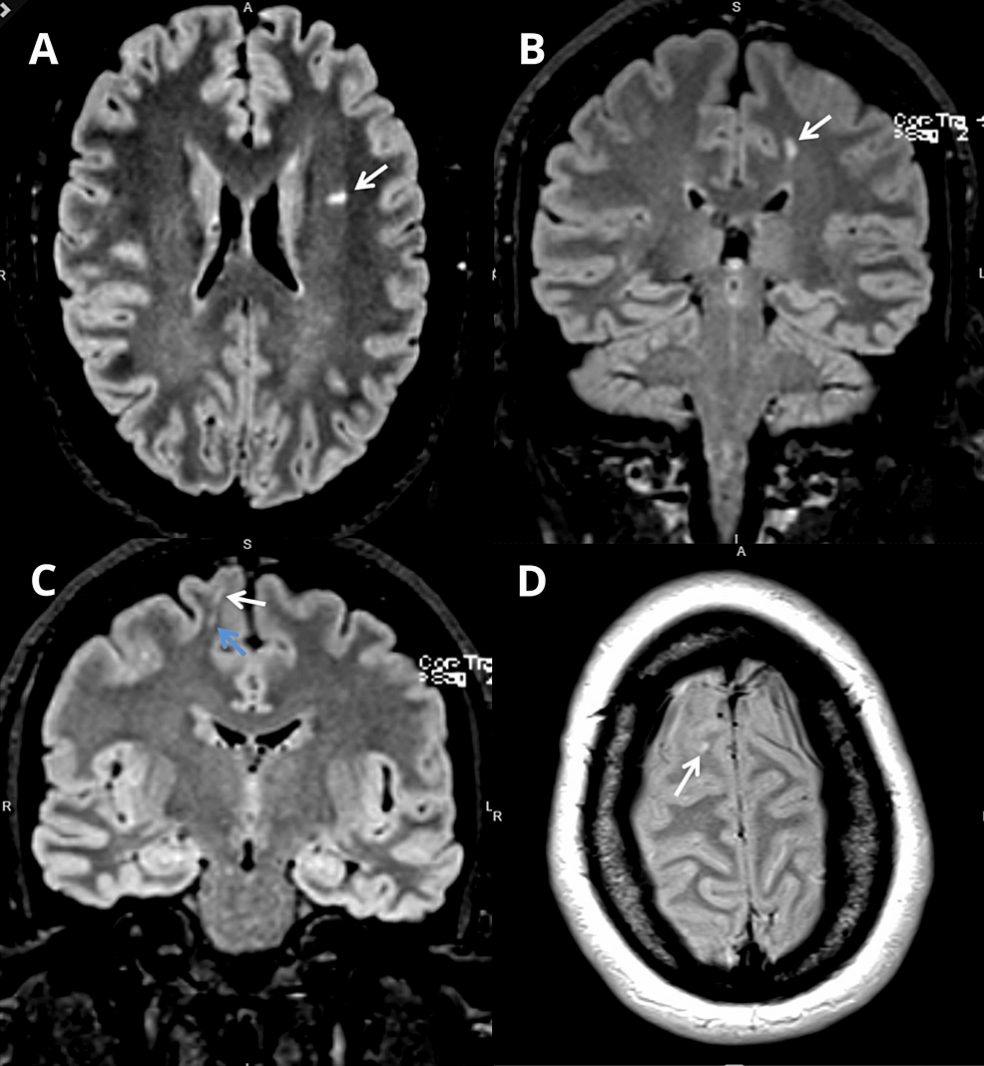
Brain MRI revealing areas of focal hypersignal in T2 (A) Brain MRI, axial T2 FLAIR, hyperintense signal in the anterior region of the left corona radiata (arrow). (B) Brain MRI, coronal T2 FLAIR, hyperintense signal in the left parasagittal frontal subcortical region (arrow). (C) Brain MRI, coronal T2 FLAIR, hyperintense signal in the right juxtacortical (white arrow) and superior frontal subcortical (blue arrow) regions. (D) Brain MRI, axial T2, hyperintense signal in the right juxtacortical region (arrow).

**Figure 2:**
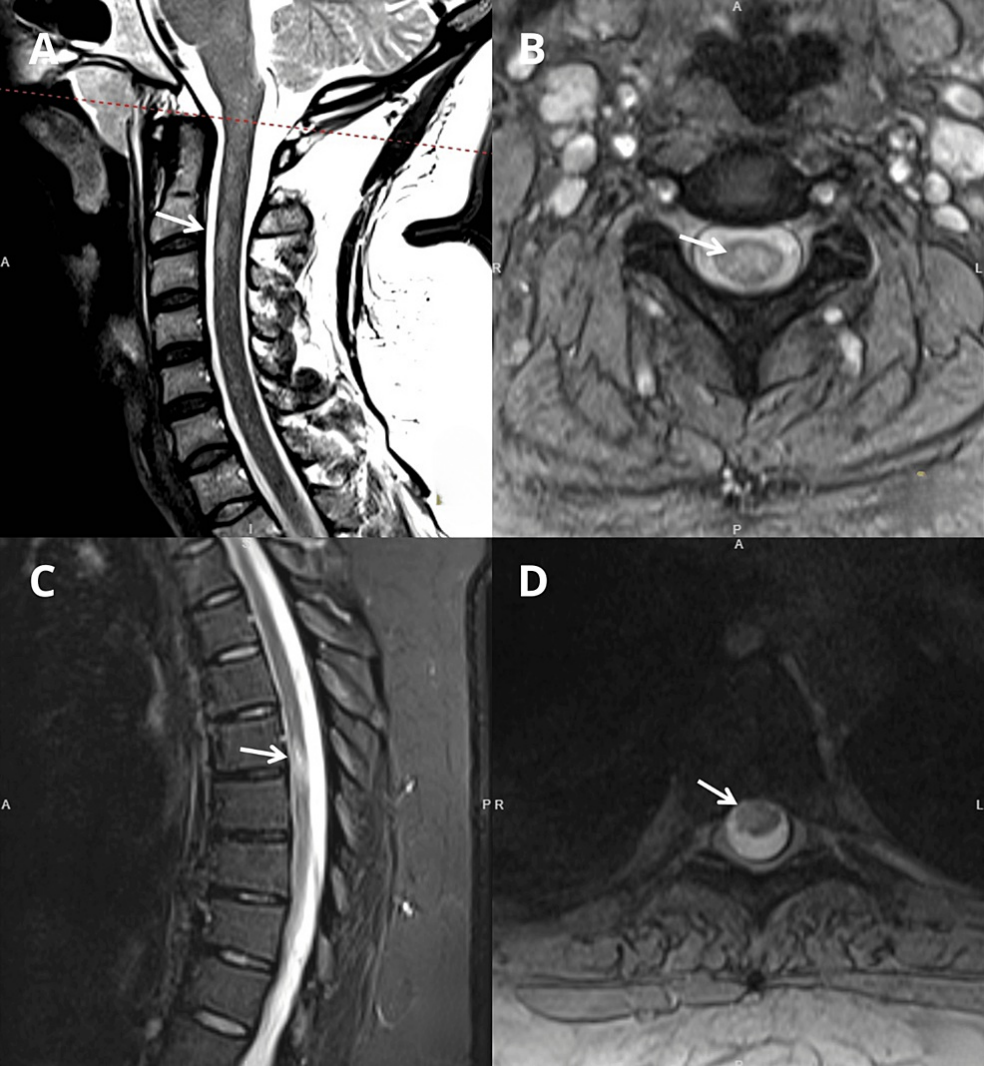
Spinal MRI revealing areas of focal hypersignal in T2 (A) Spinal MRI, sagittal T2, centromedullary hyperintense signal at C2/C3 (arrow). (B) Spinal MRI, axial T2, the same finding described in (A) (arrow). (C) Spinal MRI, sagittal T2 STIR, intramedullary hyperintense signal lateralized to the right at D6/D7 (arrow). (D) Spinal MRI, axial T2, the same finding described in (C) (arrow).

Brain MRI revealed lesions with hyperintense signals on T2 and T2 FLAIR (Figure 1). At the level of the cervical and dorsal spinal cord, there were areas of lesion with hyperintense translation in T2 and T2 STIR, with greater expression at the cervical level in C2/C3 and the dorsal level in D6/D7 and D7/D8 (Figure 2). These findings could correspond to primary demyelinating lesions of the central nervous system (CNS). In view of the above, she was referred to the neurology consultation.

One month later, at the neurology consultation, she maintained complaints of allodynia and mentioned a burning sensation on a band on the right of the mid-dorsal region. A full neurological examination was carried out without any remarkable findings. In view of the clinical picture and the MRI findings, a diagnosis of MS was considered probable. Complementary diagnostic and therapeutic tests were requested: analytical study, cerebrospinal fluid (CSF) leak study, and neuro-ophthalmology consultation. The analyses revealed a decrease in folic acid and vitamin D levels, and supplementation was started. The lumbar puncture was made difficult by her obesity (body mass index, 35) and ultimately unsuccessful.

Probable MS was assumed. Considering the patient’s preferences and upon the patient’s request, specific treatment was not started. A new MRI was carried out a year later, which yielded findings broadly similar to the previous MRI. This case was discussed at a team meeting, and the patient continues to be monitored by neurology, still without undergoing targeted treatment.

## Discussion

The clinical case presented here is of a 29-year-old who, three months after giving birth, develops vertigo, followed by blurred vision, weakness of the lower right limb, and abdominal allodynia. The diverse set of symptoms she presented with, together with the insistency of seeing the family doctor and the priors of anxiety, were obstacles to the diagnosis. As she also had a history of several visits to primary care for various complaints, somatization seemed more plausible than the de novo onset of neurological disease.

In MS, dysregulated cells of the immune system attack the myelin sheath of the nerve fibers across the CNS, causing demyelination in the brain and spinal cord. MS signs and symptoms are contingent upon the specific regions of the CNS impacted [3]. Patients experience varying levels of impairment based on the extent of inflammation and the resulting damage [3]. Some of the most common clinical manifestations include fatigue, vision problems, muscle spasms, numbness, paresthesia, limb weakness, stiffness, mobility problems, pain, depression, anxiety, bladder problems, vertigo, and constipation [3]. This seemingly inconsistent and diverse plethora of signs and symptoms may slow down and make the diagnosis difficult. Furthermore, as many times only a few symptoms are found in the same patient, it can lead to erroneous diagnoses.

The diagnosis of MS is complex and relies on a combination of clinical, imaging, and laboratory findings, as suggested by the McDonald criteria [2]. The diagnosis of MS is supported by the existence of the dissemination of MS disease characteristics in space and time [2]. Dissemination in space refers to the presence of lesions in various anatomical locations in the CNS (infratentorial, juxtacortical, cortical, periventricular, and spinal cord) [2]. Dissemination over time refers to the development of new lesions over time or the existence of multiple distinct clinical attacks [2]. In addition, in patients who present with an isolated clinical attack, the presence of specific oligoclonal bands in the CSF can also fulfill the criterion of dissemination in time [2]. In this case, the most likely diagnosis was highly hinted at by the MRI after careful anamnesis. Although there were no temporal criteria, the diagnosis of MS was considered probable, and it was deemed important to monitor its evolution and repeat the lumbar puncture procedure. Blood work is also important, if not only to eliminate other possible pathologies.

Many studies point toward an intricate interplay between genetic (MHC and non-MHC genes) and environmental factors (such as geographical and seasonal influences, diet, gut microbiome, toxins, viral exposure, vitamin D deficiency, lowered UV radiation exposure, and others), affecting both the susceptibility to developing MS and the progression of the disease [3,7].

Lane and Yadav [3] elaborated on risk factors for MS, many of which can be found in our patient, namely gender, age, race, stress (childbirth), and low serum vitamin D levels.

The first three months postpartum have a higher risk for both symptomatic MS disease activity and MRI-identifiable changes when compared with pregnancy [8]. The percentage of postpartum patients with flares varies between 14% and 30% [5,9], with hormonal changes in this period appearing to drive the increased activity [8]. Also, when pregnancy terminates, so does the status of immune tolerance in pregnancy. While its effects are more widely known toward the fetus and placenta, there may be wider-reaching implications. It is worth noting that women with more active disease before and during the pregnancy period are equally more affected in the postpartum [10]. Furthermore, pregnancy and postpartum can be stressful by themselves, and stressful life events have been pointed out as conferring an increased risk of developing new demyelinating lesions as well as increasing disease activity [6,11,12]. The triggering factor for MS in the case herein presented may have been the stress associated with childbirth.

Our patient had vitamin D deficiency, which is not uncommon in Portugal. In fact, a substantial number of Portuguese adults, according to some studies surpassing 60%, experience vitamin D deficiency [13]. Epidemiological studies have found there is a widespread deficiency of vitamin D in the general population and MS patients alike [3,14]. Numerous studies have proposed a link between vitamin D deficiency and an increased risk of MS, as evidenced by lower serum vitamin D levels observed in MS patients compared to control groups [3,14]. In women with MS before pregnancy, during pregnancy, and while breastfeeding, it is common to observe a deficiency in vitamin D [14].

A few studies (Hellwig et al. [15] and Langer-Gould et al. [16]) have indicated a potential protective function of exclusive breastfeeding in reducing the risk of relapse during the postpartum period, while other studies (Airas et al. [14] and Portaccio and Amato [10]) found no such effect.

## Conclusions

This case report highlights the diagnostic challenges encountered in a 29-year-old woman who, three months postpartum (first son), presented with a diverse array of symptoms, including vertigo, blurred vision, right lower limb weakness, and abdominal allodynia. The broad range of symptoms, the persistent pursuit of medical attention, and the priors of anxiety presented notable challenges in reaching a prompt diagnosis. The symptoms of MS can vary widely, including fatigue, muscle weakness, paresthesia in one limb, ataxia, optic neuritis, and diplopia, among others. The postpartum period is a time when women may be at greater risk of having their first MS outbreak, and this case emphasizes the need for heightened awareness in postpartum healthcare, particularly when confronted with a combination of neurological symptoms.

The family doctor played a central role in the diagnosis process as he carried out an early assessment and referentiation to secondary care. At the same time, he provided support and advice for the mother and newborn.

## References

[1] Van Der Walt A, Nguyen AL, Jokubaitis V (2019). Family planning, antenatal and post partum care in multiple sclerosis: a review and update. Med J Aust.

[2] McGinley MP, Goldschmidt CH, Rae-Grant AD (2021). Diagnosis and treatment of multiple sclerosis: a review. JAMA.

[3] Lane M, Yadav V (2020). Multiple sclerosis. Textbook of Natural Medicine.

[4] Noonan CW, Kathman SJ, White MC (2002). Prevalence estimates for MS in the United States and evidence of an increasing trend for women. Neurology.

[5] Hughes SE, Spelman T, Gray OM (2014). Predictors and dynamics of postpartum relapses in women with multiple sclerosis. Mult Scler.

[6] Djelilovic-Vranic J, Alajbegovic A, Tiric-Campara M, Nakicevic A, Osmanagic E, Salcic S, Niksic M (2012). Stress as provoking factor for the first and repeated multiple sclerosis seizures. Mater Sociomed.

[7] Baecher-Allan C, Kaskow BJ, Weiner HL (2018). Multiple sclerosis: mechanisms and Immunotherapy. Neuron.

[8] Batista S, Silva AM, Sá MJ (2018). Recommendations about multiple sclerosis management during pregnancy, partum and post-partum: consensus position of the Portuguese multiple sclerosis study group and the Portuguese Society of Obstetrics and maternal-fetal medicine. Acta Med Port.

[9] Vukusic S, Hutchinson M, Hours M, Moreau T, Cortinovis-Tourniaire P, Adeleine P, Confavreux C (2004). Pregnancy and multiple sclerosis (the PRIMS study): clinical predictors of post-partum relapse. Brain.

[10] Portaccio E, Amato MP (2019). Breastfeeding and post-partum relapses in multiple sclerosis patients. Mult Scler.

[11] Mohr DC, Goodkin DE, Bacchetti P (2000). Psychological stress and the subsequent appearance of new brain MRI lesions in MS. Neurology.

[12] Mohr DC (2007). Stress and multiple sclerosis. J Neurol.

[13] Duarte C, Carvalheiro H, Rodrigues AM (2020). Prevalence of vitamin D deficiency and its predictors in the Portuguese population: a nationwide population-based study. Arch Osteoporos.

[14] Airas L, Jalkanen A, Alanen A, Pirttilä T, Marttila RJ (2010). Breast-feeding, postpartum and prepregnancy disease activity in multiple sclerosis. Neurology.

[15] Hellwig K, Rockhoff M, Herbstritt S (2015). Exclusive breastfeeding and the effect on postpartum multiple sclerosis relapses. JAMA Neurol.

[16] Langer-Gould A, Smith JB, Albers KB (2020). Pregnancy-related relapses and breastfeeding in a contemporary multiple sclerosis cohort. Neurology.

